# Perturbations in Lineage Specification of Granulosa and Theca Cells May Alter Corpus Luteum Formation and Function

**DOI:** 10.3389/fendo.2019.00832

**Published:** 2019-11-29

**Authors:** Mohamed A. Abedel-Majed, Sarah M. Romereim, John S. Davis, Andrea S. Cupp

**Affiliations:** ^1^Department of Animal Production, School of Agriculture, University of Jordan, Amman, Jordan; ^2^Department of Biological Systems Engineering, University of Nebraska-Lincoln, Lincoln, NE, United States; ^3^Department of Obstetrics and Gynecology, Olson Center for Women's Health, University of Nebraska Medical Center, Omaha, NE, United States; ^4^VA Nebraska-Western Iowa Health Care System, Omaha, NE, United States; ^5^Department of Animal Science, University of Nebraska-Lincoln, Lincoln, NE, United States

**Keywords:** follicle, granulosa cell, theca cell, corpus luteum, infertility, differentiation, PCOS, androgen

## Abstract

Anovulation is a major cause of infertility, and it is the major leading reproductive disorder in mammalian females. Without ovulation, an oocyte is not released from the ovarian follicle to be fertilized and a corpus luteum is not formed. The corpus luteum formed from the luteinized somatic follicular cells following ovulation, vasculature cells, and immune cells is critical for progesterone production and maintenance of pregnancy. Follicular theca cells differentiate into small luteal cells (SLCs) that produce progesterone in response to luteinizing hormone (LH), and granulosa cells luteinize to become large luteal cells (LLCs) that have a high rate of basal production of progesterone. The formation and function of the corpus luteum rely on the appropriate proliferation and differentiation of both granulosa and theca cells. If any aspect of granulosa or theca cell luteinization is perturbed, then the resulting luteal cell populations (SLC, LLC, vascular, and immune cells) may be reduced and compromise progesterone production. Thus, many factors that affect the differentiation/lineage of the somatic cells and their gene expression profiles can alter the ability of a corpus luteum to produce the progesterone critical for pregnancy. Our laboratory has identified genes that are enriched in somatic follicular cells and luteal cells through gene expression microarray. This work was the first to compare the gene expression profiles of the four somatic cell types involved in the follicle-to-luteal transition and to support previous immunofluorescence data indicating theca cells differentiate into SLCs while granulosa cells become LLCs. Using these data and incorporating knowledge about the ways in which luteinization can go awry, we can extrapolate the impact that alterations in the theca and granulosa cell gene expression profiles and lineages could have on the formation and function of the corpus luteum. While interactions with other cell types such as vascular and immune cells are critical for appropriate corpus luteum function, we are restricting this review to focus on granulosa, theca, and luteal cells and how perturbations such as androgen excess and inflammation may affect their function and fertility.

## Introduction to the Development and Function of the Ovary

The ovarian follicle is the structural and functional unit of the mammalian ovary (1–3), which provides the necessary environment for oocyte growth and maturation (1, 3). The corpus luteum, which is derived from the somatic cells of a follicle after the oocyte has been released during ovulation, is a crucial part of pregnancy. Any defects in ovarian function have a negative effect on female reproductive health and fertility (4). In order to fully understand the development of an ovarian follicle and the subsequent formation of the corpus luteum (CL), it is helpful to know the origins of the cells of the ovary. Much of the information provided in this review is based on observations from studies in the bovine with references to human and mouse ovarian development.

### Ovarian Development

The assembly of ovarian follicles is the developmental process that mediates the formation of primordial follicles containing an individual oocyte (3, 5–7). Oocytes originate as primordial germ cells (PGC) from the endoderm of the embryonic sac (3, 7). The PGCs migrate by amoeboid movement from the epithelium of the yolk sac into the hindgut and arrive at the gonadal ridges (undifferentiated gonads) (3, 8–10). The PGCs undergo a limited number of mitotic divisions during their passage and upon arrival at the gonadal ridge (10). The gonadal ridges develop as a thickening of the coelomic epithelium on the mesonephros (temporary embryonic kidneys) and make connections with mesonephric tissue by streams of cells called rete-ovarii (3). When PGCs migrate they form oogonial clusters or cysts within the gonadal ridge (6, 11, 12). Mitosis of the oogonia peaks and then ceases during embryonic development, and that early population boom determines the number of germ cells available to all female mammals. Subsequently, the oogonia enter meiosis (8). Following initiation of the meiotic process, the oogonial germ cells enlarge (10) (mice). The meiotic process then leads the oogonial clusters or cysts to break apart to become oocytes surrounded by a single layer of squamous pre-granulosa cells to form primordial follicles (6, 8, 11, 13) (mice and bovine) which establish the ovarian reserve.

### Follicle Maturation

**Figure 1:**
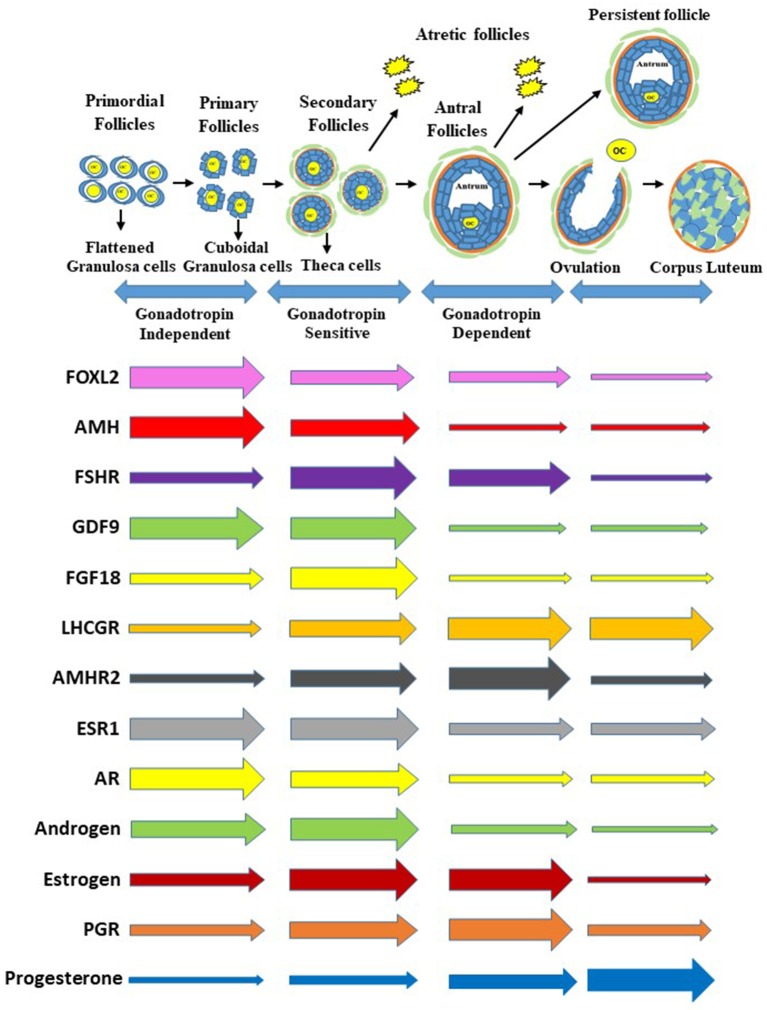
Process of follicle progression from primordial, primary, secondary, and antral follicles through gonadotropin independent, gonadotropin sensitive, and gonadotropin dependent phases to ovulation, development of a persistent follicle, follicular atresia and formation of corpus luteum. Expression of growth factors, hormone receptor genes, and steroids and steroid receptors during each stage of folliculogenesis. The gene expression is representative of the whole follicle or corpus luteum contribution and not individual cells. Furthermore, the size of the arrows at the beginning are not relative to each gene above or below but relative to the horizontal changes through the follicle stages and formation of the corpus luteum.

The initiation of follicle growth involves the transition of the primordial follicle from the quiescent follicle pool to the growing pool (transitional, primary, secondary, and tertiary) (11, 14, 15) (Figure 1). Morphological changes in granulosa from squamous/flattened (less differentiated) to cuboidal epithelial granulosa cells are the histological evidence for the transition from primordial to primary follicles (16, 17) (Figure 1). The growth of an oocyte starts immediately after the activation of the primordial follicle. Further, Braw-Tal and Yossefi (16) reported as the number of granulosa cells layers around the oocyte increased there was a corresponding enlargement of the size of oocyte.

The cuboidal granulosa cells in the primary follicle then start to proliferate, transitioning to a secondary follicle which is characterized by multiple layers of granulosa cells surrounding an oocyte (18, 19) (Figure 1). When multiple granulosa cell layers have formed in the mammalian ovary, a layer of theca cells differentiate to surround the granulosa cells outside of the basement membrane (14). This layer of theca cells will eventually become two distinct types of theca cells, the inner theca interna and the outer theca externa. The theca interna is a layer of highly vascularized steroidogenic cells, while the theca externa is a loosely organized band of non-steroidogenic cells (20).

Finally, a tertiary (or antral) follicle is formed and is characterized by the presence of a fluid-filled cavity known as an antrum (18, 19) (Figure 1). The mural granulosa cells that line the wall of the follicle are critical for steroidogenesis and ovulation; while cumulus granulosa cells surround the oocyte and promote its growth and development (18). Greater numbers of antral follicles present in the ovary are indicative of increased ovarian reserve and are associated with increased fertility in heifers (21).

As an antrum forms, tertiary follicles continue to grow and develop multiple granulosa and theca cell layers (Figure 1). The granulosa and theca cells are a site of action for the gonadotropins and a site for production of steroid hormones (22). An increase in gonadotropin releasing hormone from the hypothalamus stimulates gonadotropin release from the pituitary, which promotes follicular growth and estrogen (E2) secretion from the ovaries (23).

### Follicle Waves and Ovulation

Receptors for the gonadotropin follicle stimulating hormone (FSH) are localized in the granulosa cells of growing follicles (19, 24). An increase in FSH concentrations during the follicular phase of the reproductive cycle is very important for the development of follicular waves (2–3 waves in cattle and humans), the formation of antral follicles, and the selection of a dominant follicle (the only follicle that keeps growing while other follicles regress and undergo atresia) (25, 26) (Figure 1). The dominant follicle grows faster than the rest of the cohort, is more sensitive to FSH, and produces higher levels of estrogen and the regulatory hormone inhibin (2, 19, 27) (Figure 1).

Inhibin and estrogen concentrations in the follicular fluid of the dominant follicle have a direct negative feedback effect on FSH release at the anterior pituitary during the mid-follicular phase (2, 19, 28). The granulosa cells of the dominant follicle ultimately acquire LH receptors, a hallmark of a preovulatory follicle (Figure 1). Furthermore, increased follicular estradiol production stimulates (Figure 1) secretion of the pituitary gonadotropin luteinizing hormone (LH) and eventually induces the LH surge (18, 23). When FSH decreases and LH pulse frequency increases, the dominant follicle becomes more sensitive to LH receptors (LH/CGR) located in both the granulosa and theca cells (19). The LH surge is important in the final maturation and ovulation (follicle rupture) of the dominant follicle (18, 28), as well as in the terminal differentiation of the remaining granulosa and theca cells to form the corpus luteum. The corpus luteum is a transient endocrine structure responsible for secreting progesterone, which is required for preparing the uterus for implantation and maintenance of pregnancy (18).

### Corpus Luteum Formation and Function

After ovulation, the somatic cells (theca and granulosa) differentiate into the small luteal cells (SLC) and large luteal cells (LLC), respectively. The development of the corpus luteum into an operational endocrine gland involves growth and development of the steroidogenic cells. Optimal function of the corpus luteum requires the development of an extensive capillary network to deliver nutrients for the production and secretion of the large amounts of progesterone required to preserve pregnancy in most mammalian species. There are different morphological, physiological, and biochemical characteristics of large and small luteal cells which may reflect different follicular lineages with separate embryological origins (3, 4, 29, 30).

Bovine, ovine and human corpora lutea have two distinct steroidogenic cells with different abilities to produce progesterone (31). During differentiation, both luteal cell types acquire high levels of the key enzymes cholesterol side-chain cleavage P450 (Cytochrome P450 family 11 subfamily A1 or CYP11A1) and 3β-hydroxysteroid-dehydrogenase (3β-HSD or HSD3B) that are required to convert cholesterol into pregnenolone and then progesterone, respectively (32). The activity of these enzymes appears to be present in excess amounts in both small and LLC and does not appear to rate-limiting. The limiting step appears to be the delivery of cholesterol to mitochondria within each cell. The SLC respond to LH with large increases in progesterone secretion, and LLC have an elevated basal rate of progesterone secretion and respond to LH with a modest increase.

The luteal cells of women, monkeys, and rodents have variable responses to LH (30) which may be based on their lipid droplet morphology. Bovine (33) and ovine (34) SLC have larger lipid droplets and the LLC have abundant, dispersed small lipid droplets. The cellular origin of the large luteal cell has generally been ascribed to the granulosa cells in both ruminants and primates. Luteinization of granulosa cells gives rise to cells with cellular features and mRNA expression that are similar to the large luteal cell, whereas luteinization of theca cells gives rise to cells with features like the small luteal cell (35, 36). The origin of the two cell types is observed in the primate based on morphological evidence demonstrating portions of the corpus luteum that are primarily granulosa-lutein cells and other areas that primarily contain smaller theca-lutein cells (37).

## Lineage Specification of Granulosa and Theca Cells

### Granulosa Cell Lineage

**Figure 2:**
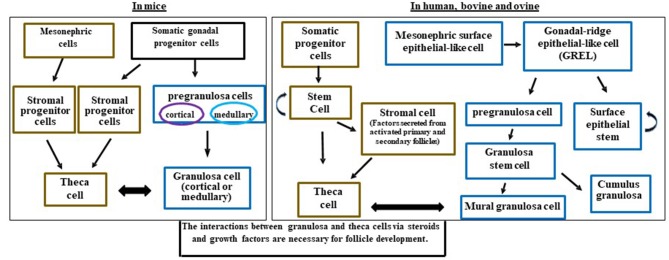
Schematic diagram illustrating the potential and known granulosa and theca cells lineages in the mice, human, bovine, and ovine ovaries and how they interact for follicle development. Somatic progenitor cells have origins from the developing coelomic gonadal epithelium and mesonephric cells. This figure was developed from information in Hummitzsch et al. (3, 41) and Rotgers et al. (42).

**Figure 3:**
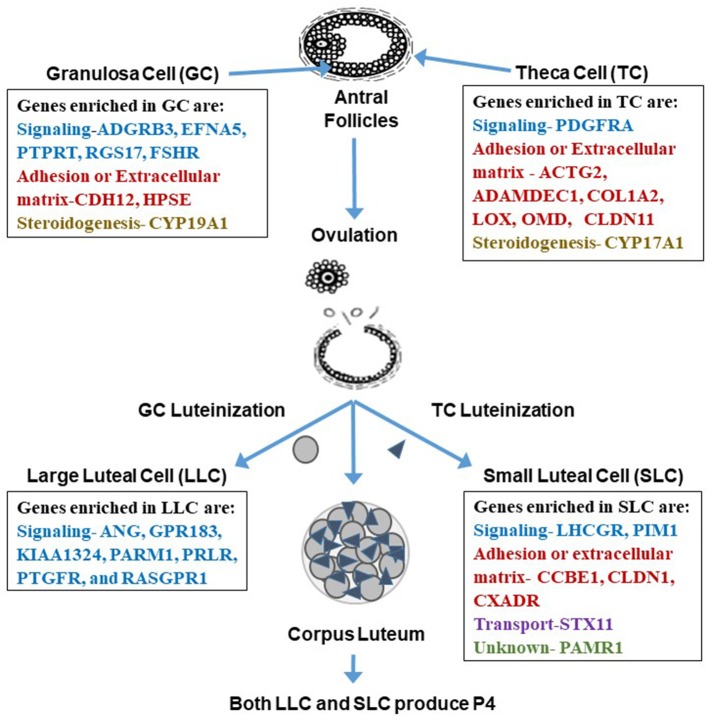
Genes enriched in granulosa cells, theca cells, large luteal cells (LLC), and small luteal cells (SLC). Enriched genes are involved in processes related to signaling, adhesion, extracellular matrix, steroidogenesis, transport, or are unknown. Interestingly, it appears that the categories change from somatic to luteal cells with mainly genes involved in signal transduction enriched in LLC and more adhesion or extracellular matrix genes enriched in SLC. Tables of these enriched genes are in Romereim et al. (36).

Granulosa cells are interesting in that they are not a uniform population of cells. Some granulosa cells have properties of stem cells including the ability to divide and form colonies (38), divide without contact inhibition (39), and may share properties of endothelial cells (40). All of these may be different depending on the species of reference. During the development of the ovary, granulosa cells may originate from several cell types (Figure 2). Early researchers provided evidence that murine granulosa cells developed from both the coelomic epithelium that forms the ovary as well as cells that migrated in from the mesonephros (43, 44). With the advent of conditional knockout mice and fluorescent tags, many of the cell lineages have been refined in the mouse model with further cell lineage differences determined in larger mammals (4, 36). The multiple cellular origins of granulosa cells (where they originate- see Figures 2, 3) may result in altered function and differentiation of these cells later during follicle formation (Figure 2) which would affect subsequent corpus luteum development and function upon ovulation. For instance, pregranulosa cells in the bovine (and potentially ovine and human) are derived from gonadal-ridge epithelial-like (GREL) cells. Also stem cells are derived from GREL cells. It is not known if stem cells can also form granulosa cells or different populations of granulosa cells such as mural and cumulus cells. Environmental factors can affect the GREL cells, which may determine if they produce stem cells or pregranulosa cells (3). If the ovarian environment fluctuates based on alterations in cytokines, growth factors, gonadotropins, or steroids it is possible that these granulosa cells might differentiate into populations with different gene expression profiles and function. Thus, further differentiation (luteinization) of these granulosa cells following ovulation may alter the luteal phenotype and also affect production of progesterone and the ability to maintain pregnancy.

The granulosa cells in mice originate from somatic progenitor cells that come from the coelomic epithelium of the gonad (4, 45, 46). These somatic progenitor cells commit to either pre-granulosa cells or stromal progenitor cells (Figure 2) (4, 46). The pregranulosa cells may have different functions and some may not proliferate depending on whether they are medullary or cortical. The medullary cells develop in the initial waves of pregranulosa differentiation and then migrate to and become part of the medullary follicles. In a second wave of pregranulosa cell differentiation, the cortical follicles develop and expand to become the ovarian reserve. A review on these different medullary vs. cortical follicles and the specific markers that differentiate them are presented in detail below (42).

In mice, cortical and medullary granulosa cells (Figure 2) commit to different types of pregranulosa cells depending on expression of specific genes. Medullary granulosa cells express the markers *Gata4*^+^*/Cdkn1b*^+^, while cortical pregranulosa cells express *Gata4*^+^*/Lgr5*^+^. The receptor for RESPO1, LGR5, is responsible for continuing development of these granulosa cells. At later stages, the cortical granulosa cells acquire *Cdkn1b*^+^, and as mature cells both cortical and medullary granulosa cells express *FoxL2*, which is critical for maintaining their granulosa-like phenotype. Without *FoxL2* these granulosa cells could revert to a testis (Sertoli-like) or epithelial lineage and express *Sox9*; this also occurs in some granulosa cell tumors (47). Expression of *FoxL2* is dependent on estrogen; thus, steroid environments lacking adequate estrogen may also affect granulosa cell function, proliferation, and development (42).

In larger mammals, the main body of evidence indicates that granulosa cells originate from the mesonephric surface epithelial cells (the temporary embryonic kidney) (Figure 2) such as in humans (42) and bovine (3, 41). Mesonephric surface epithelial-like cells break down to form GREL cells that differentiate into pre-granulosa cells and stem cells. The pre-granulosa cells then differentiate into granulosa cells. It is not known if the stem cell population in addition to self-renewal can also form a population of granulosa cells or other cell types in the ovary (Figure 2). GREL cells appear to be located above the mesonephric surface epithelium or to break into this layer and to expand. Thus, the GREL cells may also be contributing to surface epithelial cells, which repair wounds that occur due to ovulation (3, 41, 42, 45, 46). In larger species such as bovine, the pre-granulosa cells also form granulosa “stem” cells which can differentiate into cumulus and mural granulosa cells. The mural granulosa cells are the cell type that later will luteinize into the LLCs because they are the only granulosa cells expressing the LH receptor. Constant communication between granulosa cells and their surrounding environment allows for differentiation, gene expression, growth factor secretion, and cell fate (42).

### Theca Cell Lineage

There are many important roles for theca cells in the follicle including crosstalk with granulosa cells for synthesis of androgens and estrogens, as well as providing structural support of the growing follicle as it progresses through the developmental stages to produce a mature and fertilizable oocyte (42, 48). The origins of theca cells have not been definitively identified (4). Some investigators hypothesize that theca cells come from mesonephric cells in mice, humans, and bovine (3, 4, 42, 45, 48, 49) (Figure 2).

Other investigators have suggested that theca cells in the mouse originate from the stratified medial aspect of the mesonephric kidney, as they have observed cells with elongated nuclei and an overall appearance of fibroblasts (45, 46). In more recent mouse studies using fluorescent tags to *Wt1* (WT1 transcription factor) and *Gli1* (GLI Family Zinc Finger 1) genes, investigators reported that theca cells come from two sources including ovary-derived *Wt1*+ cells and mesonephros-derived *Gli1*+ cells (4). The expression of Gli1 in theca progenitor cells is induced by the paracrine signals Desert hedgehog (Dhh) and Indian hedgehog (Ihh) from granulosa cells (4). Moreover, Growth differentiation factor 9 (GDF9) from the oocyte induces Dhh/Ihh in granulosa cells (4). In the absence of Dhh and Ihh, ovaries have reduced layers of theca cells around the follicle, decreased steroid production, disrupted folliculogenesis, and fail to form corpora lutea (4). Thus, the recruitment of theca cells from the stroma is regulated by factor(s) produced by granulosa cells at the primary and secondary follicle stages (4, 50).

A previous study in women also demonstrated that theca cells originate from the coelomic epithelium of the gonadal primordium and the neighboring mesonephros under paracrine regulation from granulosa cells (51). Other studies, including those in the bovine, demonstrated that theca cells are recruited from surrounding stromal tissue by factors secreted from an activated primary follicle (3, 41, 49) (Figure 2). The actual factors have not been fully identified, but granulosa and oocyte interactions with stroma may cause stroma cells to differentiate into theca cells. It has been proposed that stroma stem cell niches exist in mice, human, and bovine ovaries that allow multiple cell types to be developed depending on the growth factors present (3, 4, 42, 48, 49, 52). Large areas of the ovary including groups of stromal cells surrounding some large blood vessels stain positively for chondroitin/dermatan sulfate epitopes (antibodies 7D4, 3C5, and 4C3) similar to stem cell niches observed in other tissues. These stromal cells may therefore represent a stem cell niche (52). Another study suggested that thecal stem cells are present in mouse ovaries (49). However, these data require further support including determination of whether all the cells or only a proportion of the cells in these stem-cell colonies express genes in the Dhh/Ihh pathway such as *Ptch1* and *2, Gli 2* and *3* (49), which would suggest a theca stem cell lineage.

## The Steroidogenesis Timeline and Pathways in Granulosa and Theca Cells

The granulosa and theca cells are a site of action for the gonadotropins (hormones secreted by the pituitary gland that act on the gonads) and a site for production of steroid hormones (22, 27, 53). Steroid hormone secretion by ovarian tissues is tightly regulated and crucial to the coordination of reproductive cyclicity (29). Steroidogenesis is the process involving the conversion of cholesterol to androgens, estradiol, and progesterone through a variety of steroid hormone intermediates (54, 55). The enzymes and series of intermediates used in humans and ruminants are collectively called the Δ^5^ pathway; whereas steroidogenesis in rodent and pig ovaries can occur through either the Δ^5^ or the Δ^4^ pathway (24). Granulosa cells and theca cells must act together for ovarian steroidogenesis. The first evidence of both theca and granulosa cells interacting to produce estradiol was reported by Falck (56) indicating estradiol production in rat follicles required both cell types. Ovarian steroid production is critical for normal ovarian processes including follicle growth, differentiation, oocyte maturation, and ovulation (57–59). Ovarian steroids are also required for the normal function and development of several tissues in the female including the uterus, breasts, skeleton, and others (54, 55, 57).

### Steroidogenesis in the Developing Ovary

The timing of steroidogenesis in the theca and granulosa cells begins surprisingly early in development. Cells within the mammalian fetal ovary already express steroidogenic enzymes and receptors and have the potential to produce both estrogen and progesterone (60–65). However, because estrogen and progesterone have been demonstrated to inhibit primordial follicle activation (5, 63, 66) and progesterone inhibits follicle assembly *in vitro* (7, 67), ovarian factors must inhibit steroidogenesis to allow these developmental events to occur. Production of estrogen and progesterone by the fetal ovary occurs early in development until the formation of germ cell cords, and then steroidogenesis is inhibited to allow follicle formation/assembly and development of the ovarian reserve (7, 63, 68).

A recent study by da Silva (69) demonstrates that fibroblast growth factor 18 (FGF18) produced by ovigerous cords in the fetal bovine ovary may inhibit production of estrogen during this early development period. FGF18 expression increases at the time when fetal ovarian estrogen and progesterone are declining, which occurs around the time of follicle assembly. Interestingly, treatment of cultures bovine fetal ovarian explants with FGF18 inhibits production of CYP19A1 and CYP11A1, causing reductions in secretion of both estrogen and progesterone *in vitro* (69). Thus, steroid production occurs early in the ovary from the ovigerous cords, which later become oogonial clusters or cysts; and FGF18 may be a paracrine growth factor that allows for both follicle development and granulosa/theca cell differentiation.

### Steroidogenesis During Follicle Maturation and Regulation by Gonadotropins

For estrogen synthesis to occur, cholesterol must first be converted to androgen in theca cells through actions of luteinizing hormone (LH), and androgens are further converted in granulosa cells to estrogens via follicle stimulating hormone (FSH) (27, 57–59) (Figure 1). In general, the binding of LH to its receptors on theca cells leads to the conversion of cholesterol into androgens like androstenedione; the androgens then diffuse into the granulosa cells to be converted to estrogens like estradiol by aromatase activity under the regulation of FSH (27, 58) (Figure 1). More specific descriptions of each step of the pathway detailing the enzymes and steroid intermediates involved are described below.

Theca cell formation occurs when a maturing follicle contains two or more layers of granulosa cells and coincides with the follicle becoming responsive to LH (48, 70) (Figure 1). Binding of LH to its receptor (LHCGR) on theca interna cells leads to signal transduction via G protein-coupled receptors (Gα_s_). Activation of Gα_s_, in turn, stimulates adenylyl cyclase, leading to an elevation in intracellular cAMP levels and subsequent activation of protein kinase A (PKA) (71). This elevation in cAMP and PKA activity promotes steroidogenesis by increasing expression of steroidogenic acute regulatory protein (STAR) as well as by increasing STAR activity via phosphorylation on serine 195 (71). Cholesterol is transported from the cytosol into the inner mitochondrial membrane by STAR in theca cells (57, 72, 73). The movement of cholesterol from the outer to the inner mitochondrial membrane by STAR is believed to be a rate-limiting step in gonadal and adrenal tissues (55, 57, 72).

The granulosa cell expresses receptors for FSH (27, 57). The binding of FSH to its receptor leads to an increase in intracellular cAMP levels and subsequent activation of several pathways including protein mitogen-activated protein kinases (p42/p44 MAPK and p38-MAPK) and phosphatidylinositol 3-kinase (PI3K), which regulate many FSH target genes such as the steroidogenic enzyme *CYP11A1* and *STAR*. In addition, PKA is activated and stimulates the phosphorylation of other regulatory proteins (CREB, β-catenin, AKT, MKP3/DUSP6, p42/44 MAPK, GSK-3ß, FOXO1, and YAP) involved in granulosa cell differentiation and steroidogenesis (27, 74–79).

The timing and magnitude of the expression of genes important to the steroidogenic process is dynamic and controlled by follicle maturation. Expression of *STAR, CYP11A1, CYP17A1*, and *HSD3B*, and *LHCGR* mRNA is present in preantral follicles (80, 81). Furthermore, expression of these steroidogenic enzymes in theca cells increases as antral follicle growth continues (81). Similar to theca cell steroidogenic mRNA expression, abundance of steroidogenic enzymes in the granulosa cells (P450 aromatase and 17β-HSD) increases as follicles progress from small to large antral stages (80). As follicles mature and near ovulation, large quantities of LH actually decrease the enzymatic activity that promotes estrogen production and instead increase enzymatic activity promoting progesterone production (24) (Figure 1).

Thus, conversion of cholesterol to androgens resulting in estrogen requires specific cells types and enzymes within the somatic compartment in the follicle. If these enzymes are over-expressed or not expressed, then this may lead to many different disorders. Excess androgen production is a criterion used to diagnose women with polycystic ovary syndrome (PCOS) (15), a metabolic and reproductive disorder. While the etiology of the PCOS phenotype is not completely known, inappropriate regulation of steroid enzymes or differentiation of somatic cells and inflammation may contribute to this disorder.

## Genes Expressed and Enriched in Follicular Somatic Cells and may be Markers for Theca and Granulosa Cells

Several genes enriched in the granulosa cell transcriptome were identified as granulosa cell gene markers when compared to theca, LLCs, and SLCs including signaling molecules: Adhesion G Protein Coupled Receptor B3 (*ADGRB3)*, Ephrin-A5 (*EFNA5*), PREDICTED-Protein Tyrosine Phosphatase receptor Type T (*PTPRT*), Regulator of G-Protein signaling 17 (*RGS17*), and Follicle Stimulating hormone receptor (*FSHR)* (Figure 3). Additional genes enriched in granulosa cells are involved in cell adhesion and extracellular matrix: Cadherin 12 (*CDH12*), and Heparanase (*HPSE);* and steroidogenesis: Cytochrome P450 family 19 subfamily A polypeptide 1 (*CYP19A1*), the critical enzyme activated by downstream signaling of FSHR to elicit conversion of androgens to estrogen (36, 82). There were also many effector molecules upregulated specifically in granulosa cells related to cell proliferation, survival, DNA replication and repair, and microtubule/chromosome rearrangement including SMADs, PLC, kinases involved in signaling cascades like MAPK3K5, and especially G-protein signaling modulators (36). Moreover, granulosa cells have their own gene expression patterns related to cell functions: cell cycle progression (genes involved in S phase and G2 phase), cellular colony formation (related to proliferation and cellular adhesion), RNA decay, functions associated with G protein and tyrosine kinase receptors, and genes related to FSH signaling protein complex assembly (36).

Genes enriched in theca cells that may be used as markers are genes involved in signaling: Platelet-derived growth factor receptor, alpha polypeptide (*PDGFRA)*; cell adhesion or extracellular matrix development: actin gamma 2 (*ACTG2)*, A disintegrin and metalloprotease domain-like decysin 1 (*ADAMDEC1*), Collagen type I alpha 2 chain (*COL1A2)*, Lysyl Oxidase like 1 (*LOXL1)*, Osteomodulin *(OMD)*, Claudin 11 (*CLDN11)*, and steroidogenesis: Cytochrome P450 Family 17 Subfamily A Member 1 *(CYP17A1)* (36, 82) (Figure 3). Theca cells have increased gene expression patterns related to metabolism, glycolysis, oxidation of proteins, metabolism of heme, synthesis of carbohydrates, synthesis of sterols, and increased gene expression related to extracellular matrix genes such as decorin, collagens, elastin, and fibrillin (36). Interestingly, in women diagnosed with PCOS, there is a thickening of the cortex of the ovary, which may suggest upregulation of extracellular matrix proteins (83). These ovaries also are more rigid. One of the genes associated with theca cells, fibrillin 3, is a glycoprotein that is associated with the extracellular matrix, and its overexpression may also be linked to the rigid nature of these PCOS ovaries (84).

Genes enriched in granulosa cells vs. theca are mainly those regulating signaling and proliferation of cells in the follicular compartment (36). Furthermore, the expression of *FSHR* is specific to granulosa and not theca whereas *LHGCR* or *LH* is expressed in both theca and granulosa cells (85). Theca cells are enriched in genes that are involved in extracellular matrix and cell adhesion, suggesting theca cells have a more mesenchymal phenotype with more genes regulating stromal composition. Both somatic cells expressed a cell type-specific enzyme that regulates steroidogenesis (CYP19A1 and CYP17A1 in granulosa and theca cells, respectively).

## Morphological Changes to Follicular Somatic Cells after Ovulation and Luteinization

At ovulation, the antral follicle undergoes a remarkable transformation. The somatic follicular cells (theca and granulosa) luteinize into a bloody structure called a corpus hemorrhagicum, which then undergoes maturation to a highly vascularized corpus luteum (86) (Figures 1, 3). In the bovine, the luteinization process starts as granulosa cells and theca interna cells begin to differentiate into large and small luteal cells, respectively (3, 31, 36, 87) (Figures 1, 3). Small and large luteal cells are referred to as parenchymal cells that, together with fibroblasts and endothelial cells, form the basic structure of the corpus luteum (88, 89). Recently, we reported that bovine small and large luteal cell types are highly similar based on overall gene expression profiles, with some notable differences (36). A subsequent report evaluating the differences in gene expression in SLCs and LLCs in bovine corpus luteum confirmed these findings (90).

Both SLCs and LLCs possess LH receptors and synthesize and secrete progesterone (36) (Figure 1). Although SLCs are highly responsive to LH, large luteal cells are responsible for 80% of the total progesterone produced by the corpus luteum (91). As the corpus luteum matures, it is proposed that SLCs can increase in number but not in size, whereas large luteal cells can increase in size but not in number during the normal estrous cycle (89). Studies have shown that appropriate numbers of granulosa cells in the developing follicle are critical to provide the minimum population of LLCs needed to maintain progesterone production (92). Environmental factors that affect the proliferation or fate of granulosa cells may reduce luteal cell numbers with adverse effects on progesterone production by the subsequent corpus luteum.

Progesterone targets many tissues, including the hypothalamic-pituitary axis, ovary, oviduct, uterus, cervix, vagina, and mammary gland and embryo (29). Luteal progesterone secretion serves as a local luteal cell survival factor (93), regulates the length of the estrous cycle, and is essential for maintenance of pregnancy (29, 36). Progesterone also regulates the timing of ovulation in livestock species (94). When fertilization of the oocyte and implantation are successful, maternal recognition of pregnancy results in the maintenance of the corpus luteum, which ensures pregnancy maintenance and embryo development (36, 95). Otherwise, the luteolytic cascade would be initiated with the release of prostaglandin F2α in a pulsatile pattern, causing regression of the corpus luteum (36, 95). Anti-luteolytic mechanisms such as secretion of IFNT signaling molecules from the conceptus result in gene expression changes in the LLC and SLC such as increasing expression of ISG15 (interferon-stimulated gene, 15 kDa) (95, 96). Thus, ovarian somatic cells play an essential role in embryonic fate (96).

## Changes in Gene Expression in Theca and Granulosa Cells during Differentiation to SLCs and LLCs

The process of luteinization is a dramatic change in the morphology and function of the follicle. This process is initiated by the surge of LH at the time of ovulation. Specific alterations in gene expression occur in theca cells responding to the LH surge that are compatible with a transition to a SLC phenotype including a decrease in expression of *CYP17A1*, Solute carrier family 1 member 3 (*SLC1A3*), TNF receptor-associated factor 5 (*TRAF5)*, Tetraspanin 33 (*TSPAN33)*, and Hydroxyprostaglandin dehydrogenase (*HPGD)* concurrent with increased expression of Ras homolog family member B (*RHOB)* (36, 97). There are also alterations in granulosa cell gene expression in response to the LH surge that are compatible with a transition to an LLC phenotype including loss of GC expression of Cytochrome P450 family 19 subfamily A member 1 *(CYP19A1)*, Carbohydrate sulfotransferase 8 *(CHST8)*, Hydroxysteroid 17B dehydrogenase 1 *(HSD17B1)*, Glutamate cysteine ligase catalytic subunit (*GCLC)*, Solute carrier family 35 member G1 (*SLC35G1)*, and Alanine amino transferase or Glutamic-pyruvate transaminase) (*GPT*) along with the gain of expression of Pentrax 3 (*PTX3)*, Runt-related transcription factor 2 *(RUNX2)*, periostin *(POSTN)*, Rho-related GTP-binding protein *(RND3)*, tissue inhibitor of metalloprotease *1 (TIMP1)*, Neurotensin *(NTS)*, Fos proto-oncogene *(FOS)*, and regulator of calcineurin 1 (*RCAN1)* (36, 97) (Figure 3). These differences in expression of genes also characterize granulosa cells moving from an epithelial cell type to a mesenchymal phenotype as well as a steroidogenesis transition from androgen-centric and estrogen-centric to a progesterone-centric machine, allowing for the production of large quantities of progesterone necessary to support establishment and maintenance of pregnancy.

## Luteal Cell Enriched Genes that can be used as Markers for LLCs or SLCs

In addition to its function as a steroidogenic powerhouse, comparisons of gene expression profiles among granulosa, theca and luteal cells, and bioinformatics analysis predicts that a primary function of the LLC is adhesion (binding of cells, growth of epithelial tissue, and quantity of connective tissue) which is consistent with the changes that occur during luteal formation and LLC differentiation (36). Ultrastructural studies clearly demonstrate that bovine LLCs are closely associated with endothelial cells and have a vast surface area of cellular processes that connect with those cells (98), presumptively providing for adequate nutrition and substrates for progesterone production and easy access for progesterone to enter the blood stream. Genes related to cell signaling were enriched in LLCs including receptors such as Prostaglandin E Receptor 3 (*PTGER3)*, Platelet-derived growth factor receptors *(PDGFR)*, Prolactin receptor *(PRLR)*, Fms related tyrosine kinase 1 (*FLT1)*, kinase insert domain receptor *(KDR)*, adrenergic receptor (*ADRA2B*), endothelin receptor (*EDNRB*), Transforming growth factor-beta receptor type 2 (*TGFBR2)*, and TNF receptor superfamily member 21 *(TNFRSF21)* (36) (Figure 3). Moreover, the LLC is enriched with genes coding for secreted signaling molecules including Platelet derived growth factor subunit A (*PDGFA)*, Parathyroid Hormone Like Hormone *(PTHLH)*, Angiogenin (*ANG***)**, G protein-coupled receptor 183 (*GPR183*), Prostate androgen-regulated mucin-like protein 1 (*PARM1*), Prostaglandin F receptor (*PTGFR*), and RAS guanyl releasing protein 1 **(***RASGPR1***)** (Figure 3), genes for angiogenesis including Ephrin-B2 (*EFNB2)*, Platelet-derived growth factor receptor beta *(PDGFRB)*, and Vascular *E*ndothelial *G*rowth *F*actor A *(VEGFA*), genes for differentiation of cells including Neurogenic Locus Notch Homolog Protein 3 (*NOTCH3)*, Parathyroid Hormone Like Hormone *(PTHLH)*, Transforming Growth Factor-beta receptor type 2 (*TGFBR2)*, and *WNT11*, genes for immune and inflammatory responses (chemokines, interleukins, tumor necrosis factor family molecules), genes for synthesis of lipids including Acyl-CoA Oxidase 2 (*ACOX2)*, Acyl-CoA Synthetase Long Chain Family Member 4 (*ACSL4)*, Cytochrome P450 Family 7 Subfamily B Member 1 (*CYP7B1)*, Retinol Dehydrogenase 10 (*RDH10*), and genes related to ion transport including Solute Carrier Family 7 Member 8 (*SLC7A8)*, ATPase Na+/K+ transporting subunit beta 2 (*ATP1B2*) (36). Thus, many changes occur in LLC gene expression during differentiation that aid in developing vasculature, regulation of immune cell interactions, and maintenance of progesterone production.

In contrast, as determined by genes specifically enriched in SLCs, bioinformatics revealed their primary function as metabolism, including metabolism of phospholipids, peptides, and sterols as well as regulation of the concentration of ATP (36). Genes enriched in SLCs are signaling molecules including Luteinizing hormone receptor (*LHCGR*), Pim-1 Proto-Oncogene (*PIM1*). SLCs also had three genes enriched involved in adhesion and extracellular matrix including Collagen and Calcium binding EGF (*CCBE1*), Claudin 1 (*CLDN1*), Coxsackie virus and adenovirus receptor (*CXADR*); and Syntaxin 11 (*STX11*) is involved in transport while Peptidase domain containing associated with muscle regeneration 1 (*PAMR1*) gene function is unknown (36) (Figure 3). When comparing SLC gene expression to that of LLCs, theca cells, and granulosa cells, SLCs had the greatest number of genes involved in signal transduction including the following: both receptors and ligands related to BMP signaling, complement components involved in immune response, and effector molecules such as kinases and phospholipases (36).

## Inflammation Can Affect Granulosa and Theca Cells Which May Perturb Corpus Luteum Formation and Function

Ovulation is similar to an inflammatory cytokine-mediated response (99, 100) and is associated with an increase in vasodilation, production of prostaglandin, cell proliferation, and secretion of tissue as well as local growth regulatory factors (101, 102). Some studies have suggested that the innate immune system is active in connection with ovulation (101–103). Cytokines are both key modulators of the immune system and contribute to regulation of the ovarian cycle. In fact, the expression and function of innate immune cell-related genes in non-immune cells within the ovary has been reported and provides a novel and important regulatory system during ovulation (104).

Cytokines and chemokines play an important role in ovarian function and follicular development throughout the estrous cycle. One study in early antral follicles (22) found 446 genes in granulosa cells and 248 genes in theca cells, with only 28 regulated genes that were common to granulosa and theca cells. These shared genes were associated with bovine antral follicle development, and they have identified candidate growth factors and cytokines potentially involved in inflammation and cell–cell interactions required for ovarian function such as the following genes: Macrophage inflammatory protein (*MIP1 beta*), Teratocarcinoma-derived growth factor 1 (*TDGF1*), Stromal derived growth factor 1 (*SDF1*; i.e., *CXCL12*), Growth differentiation factor 8 (*GDF8*), Glia maturation factor gamma (*GMFG*), Osteopontin (*SPP1*), Angiopoietin 4 (*ANGPT4*), and Chemokine ligands (*CCL 2, 3, 5*, and *8*) (22).

**Figure 4:**
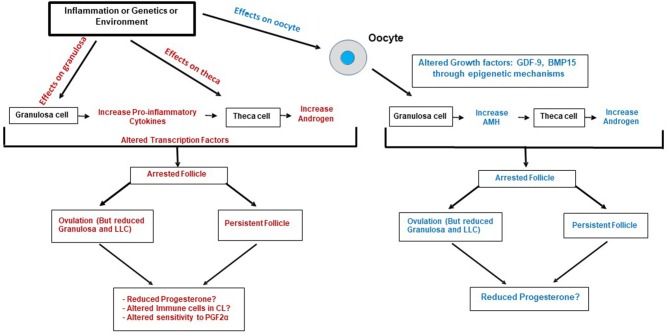
Proposed pathways where Inflammation/Genetics or Environment may cause altered function of individual cells of the follicle (granulosa, oocyte and theca) to alter transcription or expression of genes by epigenetic mechanisms resulting in altered cell to cell communication, arrested follicle development, and anovulation, altered luteal function, and female infertility.

During the latter stages of follicular development, there is cellular restructuring including follicular wall breakdown, oocyte expulsion and cellular reorganization in preparation for ovulation. These processes are initiated by pro-inflammatory cytokines and chemokines such as interleukin 1α (IL-1α), tumor necrosis factor alpha (TNFα), and chemokine (C-C motif) ligand 4 (CCL4) which stimulate migration of macrophages, monocytes, and leukocytes into the follicle (105). The cytokines indicated above induce an M1 pro-inflammatory phenotype in the infiltrating macrophages that secrete these same pro-inflammatory cytokines, producing a feed-forward mechanism required for ovulation. In some situations such as persistent follicles (i.e., arrested follicles, such as in PCOS), expression of these pro-inflammatory cytokines is enhanced and may promote increased migration of macrophages, neutrophils, and monocytes into the dominant follicle to potentially reduce granulosa cell proliferation, steroidogenesis and subsequent corpus luteum function (106, 107) (Figure 4). Inflammation and cytokines are dramatically higher in follicular fluid from follicles of PCOS patients who are obese (108). In granulosa cells during ovulation, 259 genes were found to be upregulated (2–80-fold) (102). Most of these genes were involved in cytokine signaling to initiate inflammation, innate immunity (102, 109), and cell proliferation factors involved in tissue repair during ovulation (102). The most prominent pathways that represent several modes of cytokine signaling in granulosa cells include MAPK and JAK/STAT signaling (102).

The luteolytic process has also been compared to an acute inflammatory response since it involves infiltration of neutrophils (110–113), macrophages (111, 114–117), and T lymphocytes (111, 115, 116). There is also secretion of cytokines that recruit and potentially trigger leukocytes to become active (118–120). PGF2α rapidly induces many cytokine transcripts in luteal cells (121) such as Tumor necrosis factor alpha (*TNF*) (122, 123), Transforming Growth Factor Beta 1 (*TGFB1*) (123–125), interleukin 1 beta (*IL1B*) (122, 126), and chemokines such as C-C motif chemokine ligand 2 (*CCL2*) (118, 125, 127) and C-X-C motif 8 (*CXCL8*) (112, 113, 123, 125, 126, 128). These cytokines can inhibit progesterone secretion (129–132) and stimulate PGF2α secretion (129, 130, 132). These cytokines can also induce apoptosis in luteal cells (130, 131, 133).

Luteolytic factors secreted by the uterus induce a reduction in blood flow, recruitment and migration of immune cells, reduction in progesterone production, and secretion of pro-inflammatory cytokines within the corpus luteum to induce regression (134, 135). As the intra-luteal concentrations of PGF2α and inflammatory cytokines increase, they may act within an auto-amplification loop until they eventually reach a critical point from which there is no return from the luteolytic cascade (121). Much of the research on immune cells has been conducted in the regressing or late corpus luteum. There is information to suggest that immune cells, mainly macrophages, are present in the corpus luteum (136) and that SLCs produce factors which recruit immune cells (90). Also a recent study demonstrated that depletion of immune cells prior to ovulation blocks ovulation and prevents corpus luteum formation (137).

Several papers have shown that cows with persistent or cystic follicles do have problems ovulating, and women with PCOS also have chronic inflammation. Thus, the types and numbers of immune cells in the follicle may alter ovulation and/or the function and formation of the corpus luteum (Figure 4). Lymphocytes do migrate into the corpus luteum and regulate cellular proliferation, steroidogenesis and cell differentiation; however, much needs to be determined about which specific cell types (T, B, Natural Killer; NK) these are and how they might affect any macrophages that are present at the time of ovulation (136). Poole and Pate concluded that, in bovine corpus luteum, the microenvironment regulates the recruitment or differentiation of lymphocyte cells, and these specific lymphocyte cell types (resident T cell lymphocytes) interact with steroidogenic cells to promote appropriate progesterone production and function of the developing corpus luteum.

A microarray analysis comparing SLC and LLCs determined that gene expression of immune factors were greater in SLCs compared to LLCs in bovine mid-stage corpus luteum, with upregulation of chemokines *CCL2, CCL3, CCL4, CCL5, CCL8*, and *CCL16*, and several CXC chemokines as well as *CX3CL1*. This may suggest that the theca and SLCs have critical functions in directing immune cells to the site of luteal formation to either stabilize its structure, increase progesterone, or enhance vascular development (90).

## Perturbations in Gene Expression in Granulosa or Theca Cells Through Inflammation and Altered Steroidogenesis May Alter LLCs or SLCs and Impair Luteal Function

### Stressors During Ovarian Development

Inflammation, environmental stressors, abnormal follicular somatic cell differentiation can result in excess androgens, follicular arrest, anovulation, persistent follicles, and sub-functional corpora lutea, all of which may contribute to female infertility disorders (3, 138) (Figure 4). Since steroidogenesis occurs in early fetal life, maternal stressors may perturb cell differentiation of the ovary to alter theca or granulosa cell lineage differentiation and steroid enzyme expression (60–65). Thus, excessive androgen production during the formation of the ovary may affect progenitor cells that give rise to both granulosa cells and theca cells to change their identity. Furthermore, if oocytes or granulosa cells do not express appropriate factors, theca cell differentiation may be altered which may negatively impact formation of the SLCs (and possibly LLCs through cell-cell interaction) and adversely affect the formation and function of the corpus luteum.

### Excess Androgens May Affect Follicle Growth and Proliferation of Granulosa Cells

Increased amounts of androgen hormones in the follicular microenvironment are a major factor for follicle arrest (139). Dehydrotestosterone (DHT) treatment reduces granulosa cell cycle progression and FSH-stimulated cell proliferation (140) by decreasing the expression of cell cycle proteins (141). Additionally, DHT stimulates the expression of tumor suppressor gene (*PTEN*) through peroxisome proliferator-activated receptor gamma (PPAR_**γ**_) which in turn suppresses the phosphatidylinositol 3-kinase (PI3K)/AKT signaling pathway, leading to cell cycle arrest and reducing granulosa cell proliferation and viability (142). Several researchers (15, 143) suggest that androgen excess is the most important symptom in PCOS. Women diagnosed with PCOS may have abnormal theca cell function and increased androgens, leading to abnormal ovarian follicular development (15, 144, 145). Excess circulating androgen is associated with ovarian dysfunction and metabolic disorders in women (146). Androgenized rats had fewer estrous cycles and lower numbers of mature and ovulated follicles (142, 145).

### TGFβ Family Perturbations Can Alter Granulosa Cell Function

Transforming growth factor beta family growth factors have specific roles in follicle arrest and progression. One TGFβ family member, Anti-Mullerian growth factor (AMH) is produced by large dominant follicles (in humans and bovine) and suppresses other growing follicles until just prior to ovulation (147) (Figures 1, 4), AMH also inhibits FSH-stimulated functions of bovine granulosa cells (147). A majority of women diagnosed with PCOS also have excess granulosa cell production of AMH and another TGFβ family member, follistatin (FST) (148). The excess AMH may be caused by genetic variants in the *AMH* or *AMHR* genes (149) or due to oocyte factors (BMP15 and GDF-9) causing acetylation of histone 3 lysine 27 (H3K27ac) (150) in granulosa cells to enhance *AMH* transcription (Figure 4). Furthermore, AMH inhibits aromatase and reduces estrogen production, resulting in excess androgens (151, 152). In human follicles (85) expression of *FST* is positively correlated with *AMH, FSHR*, and *AR*. Follistatin and its family member FSTL4 are upregulated in LLCs compared to SLCs (90) in the bovine corpus luteum, suggesting that TGFβ family member secretion (e.g., FST) by LLCs may continue to regulate developing follicles for the subsequent cycle. Thus, alterations in AMH and other TGFβ family members in women diagnosed with PCOS may also indicate changes in their follicles and subsequent corpus luteum function.

### Altered Follicular Development and Subsequent Luteal Function

The development and regression of the corpus luteum in ovulatory women with polycystic ovaries have been studied and compared with normally cycling women, and the corpora lutea exhibited no morphological or degenerative differences (153). However, in women with sporadic or chronic anovulation, there is reduced progesterone throughout the menstrual cycle (154) suggesting there is potential for altered luteal function. (155). Additionally, cows with persistent follicles have been demonstrated to develop sub-functional corpus luteum which cannot maintain a pregnancy (156). In androgenized sheep models, the development of PCOS-like phenotypes also results in luteal dysfunction resulting in early pregnancy failure (12). Thus, many facets of luteal function need to be evaluated, including abnormal follicular somatic cell lineage differentiation, to determine how therapies may be employed to maintain progesterone production and promote appropriate luteal formation in mammals.

In summary, anovulation is the leading reproductive disorder in mammalian females. Understanding what affects the ovulation process in order to cause anovulation is essential to improve female fertility. The formation of a functional corpus luteum relies on the appropriate proliferation and differentiation of both granulosa and theca cells. Any disruption in the crosstalk between granulosa and theca cell or differentiation of these cells types alters lineages and gene expression profiles that could negatively impact luteinization and progesterone production. Moreover, there are a number of autocrine and paracrine factors can affect the formation of the corpus luteum, e.g., growth factors, androgen excess, and inflammatory cytokines as discussed within this review. We hope that this review will spark interest and research to better understand the complex and fascinating processes used during the formation and regression of the corpus luteum.

## Author Contributions

All authors listed have made a substantial, direct and intellectual contribution to the work, and approved it for publication.

### Conflict of Interest

The authors declare that the research was conducted in the absence of any commercial or financial relationships that could be construed as a potential conflict of interest.
